# Evaluating Ma-ol-asal Syrup for Chemotherapy-induced Fatigue in Gastrointestinal Cancer Patients: A Randomized Double-blinded Placebo-controlled Clinical Trial -

**DOI:** 10.31661/gmj.v14i.3913

**Published:** 2025-12-29

**Authors:** Ata Amani, Bayazid Ghaderi, Mehdi Pasalar, Khaled Rahmani, Kiarash Zare, Thomas Rampp, Ghazaleh Heydarirad

**Affiliations:** ^1^ Traditional Medicine and Materia Medica Research Center and Department of Traditional Medicine, School of Traditional Medicine, Shahid Beheshti University of Medical Sciences, Tehran, Iran; ^2^ Cancer and Immunology Research Center, Research Institute for Health Development, Kurdistan University of Medical Sciences, Sanandaj, Iran; ^3^ Research Center for Traditional Medicine and History of Medicine, Shiraz University of Medical Sciences, Shiraz, Iran; ^4^ Liver and Digestive Research Center, Research Institute for Health Development, Kurdistan University of Medical Sciences, Sanandaj, Iran; ^5^ Shiraz Institute for Cancer Research, School of Medicine, Shiraz University of Medical Sciences, Shiraz, Iran; ^6^ Department of Internal and Integrative Medicine, Evang. Kliniken Essen-Mitte, Faculty of Medicine, University of Duisburg-Essen, Essen, Germany

**Keywords:** Fatigue, Chemotherapy, Cancer, Honey, Supportive Care, Persian Medicine

## Abstract

**Background:**

Chemotherapy-induced fatigue (CIF) is a common and debilitating side effect in cancer patients, particularly those with gastrointestinal cancers. This study explores the potential of Ma-ol-asal, a traditional Persian herbal syrup, as a holistic, supportive approach to alleviate CIF’s physical and psychological burdens.

**Materials and Methods:**

This randomized, double-blind, placebo-controlled trial involved 120 gastrointestinal cancer patients with fatigue, randomly assigned to receive 10 mL of Ma-ol-asal (compound honey syrup) or placebo thrice daily for four weeks. Fatigue was assessed with validated scales at baseline and post-intervention once, with data analyzed to evaluate efficacy.

**Results:**

After withdrawals, 42 patients per group remained. No significant demographic or lab differences were observed. Both groups had comparable scores post-treatment across all measures, with no significant differences. Adverse events, mainly nausea, vomiting, and abdominal pain, were similar. Perception of benefit varied between groups.

**Conclusion:**

Our study shows Ma-ol-asal syrup isn’t superior to placebo for chemotherapy-induced fatigue, highlighting significant placebo effects. This emphasizes the need to understand harnessing placebo responses to improve symptom management safely.

## Introduction

Chemotherapy-induced fatigue (CIF) is a pervasive and distressing side effect that significantly impacts the quality of life and treatment outcomes of cancer patients, especially those undergoing treatment for gastrointestinal cancer [1][2]. The pathogenesis of cancer-related fatigue has not been fully elucidated, and various mechanisms can play a role in its development and exacerbation [3]. Non-specific symptomatic treatment methods include education, counseling, pharmacological methods (such as brain stimulant medications), and non-pharmacological methods (such as exercise, yoga, and acupuncture), with recent secondary studies indicating that these non-pharmacological options can have a positive impact on reducing fatigue [4].

CIF is one of the most common adverse effects experienced during chemotherapy, often lasting over two weeks. It is also recognized as having the most significant and enduring influence following treatment that persists beyond the completion of chemotherapy. Observations indicate that fatigue levels tend to peak within the first 24 to 48 hours after chemotherapy administration [5]. Research indicates that CIF affects a substantial proportion of cancer patients, with prevalence rates ranging from 80% to 96% across different cancer types and treatment protocols [6][7]. This chronic fatigue can significantly impact patients' daily functioning, emotional well-being, and overall quality of life [8].

In recent years, interest has grown in exploring the potential benefits of complementary, traditional, and integrative medicine (TCIM) in alleviating the physical and emotional burdens associated with cancer-related fatigue [9][10]. Ma-ol-asal (compound honey syrup) coming from Persian medicine (PM) sources is a combination of spices including ginger, cinnamon, musk, saffron, mestaki, pepper, rosewater, and cardamom [11]. According to the principles of PM, in individuals complaining of weakness and lack of energy, the first step in treatment should focus on improving digestion and food absorption, as well as eliminating substances and wastes that contribute to weakness. The use of Ma-ol-asal as a product beneficial in the treatment of gastrointestinal diseases and expelling phlegm and thick substances (which can be a cause of weakness and fatigue) has been mentioned [12]. Comprising a blend of natural ingredients, including honey, Ma-ol-asal is believed to possess unique properties that could offer relief and support to individuals grappling with CIF. The synergy of these natural components may provide a holistic approach to addressing the multifaceted nature of CIF, encompassing both physical and psychological dimensions. Despite advancements in cancer care and treatment strategies, the management of CIF continues to pose a significant challenge to healthcare providers and patients alike. Given the high prevalence and multidimensional nature of CIF, healthcare providers and researchers recognize the importance of addressing this side effect and implementing supportive care measures to alleviate its impact on patients. In light of the growing interest in CTIM therapies and the need for innovative approaches to managing CIF, this feasibility study aimed to investigate the efficacy of Ma-ol-asal in alleviating fatigue symptoms in patients with gastrointestinal cancer.

## Materials and Methods

### Study Design

This randomized, double-blind, placebo-controlled clinical trial enrolled 120 patients who were admitted at the Oncology Clinic of Tohid Hospital in Sanandaj. Participants were selected based on eligibility criteria and provided informed consent. They were randomly assigned to either the Ma-ol-asal group or the control group. In this clinical trial, the participants consisted of 120 patients with gastrointestinal cancer undergoing chemotherapy who reported fatigue and had been receiving treatment at the Oncology and Chemotherapy Clinic of Tohid Hospital. The study period spanned from September to December 2023. The intervention lasted for eight weeks.

This study was approved (code IR.SBMU.RETECH.REC.1399.260) by the Ethics Committee of Shahid Beheshti University of Medical Sciences and registered at the Iranian Registry of Clinical Trials, with code No. IRCT20191211045693N1 in August 2023.

### Participants

Participants aged 18 to 70 years, diagnosed with gastrointestinal cancer confirmed by pathology, and undergoing chemotherapy with standard regimens were included. Eligible patients had a minimum hemoglobin level of 10 g/dL, hematocrit of at least 30%, and a Visual Analogue Fatigue Scale (VAFS) score of 4 or higher. Patients with clinical symptoms of hypothyroidism, unstable cardiovascular, hepatic, or renal conditions, diabetes mellitus, depression, anxiety disorders, or debilitating respiratory diseases were excluded. Additionally, individuals with a history of allergy to honey or its components, or any ingredient in the honey syrup, were not eligible. Those with uncontrolled health conditions, severe infections, serious comorbidities, or diagnosed psychiatric illnesses under treatment were also excluded. Pregnant women and individuals taking medications known to affect fatigue—such as monoamine oxidase inhibitors (e.g., furazolidone, isocarboxazid, meclobemide, phenelzine, procarbazine, selegiline, tranylcypromine), sympathomimetic amines (e.g., amphetamine, phenylpropanolamine, pseudoephedrine), stimulants like methylphenidate, serotonin reuptake inhibitors (e.g., fluoxetine, paroxetine, sertraline, venlafaxine, nefazodone, mirtazapine, buspirone, trazodone), tricyclic antidepressants (e.g., amitriptyline, clomipramine, desipramine, doxepin, imipramine, nortriptyline, protriptyline), serotonergic agents (e.g., lithium, mappiridine, dextromethorphan), treatments for anemia (transfusions or erythropoietin alpha), or other relevant medications, were excluded. Patients who withdrew consent after randomization, did not adhere to the medication protocol for more than three days, or whose health worsened during the study requiring additional interventions such as surgery, were also excluded.

### Sample Size

Based on the study objectives, a conservative estimate assumed a fatigue prevalence of 50% in the control group. With a significance level of 5%, a statistical power of 80%, and an effect size of 25% in the difference of the new method's efficacy compared to the control group in reducing fatigue, a sample size of 59 participants per group was calculated, totaling 118 participants.

### Preparation of Ma-ol-asal and Placebo

The Ma-ol-asal syrup was purchased from Niak Pharmaceutical Company, Gorgan, Iran, under the marketing authorization number S-94-0425, and is based on a traditional recipe from ancient pharmacopoeia [13]. Its pharmaceutical components, such as probiotics, and its properties—including immune system boosting, antibacterial, and antioxidant effects—have been explored in recent studies [14][15]. A placebo syrup was also obtained from the same company, prepared in bottles identical in appearance to Ma-ol-asal to ensure blinding. The placebo consisted of water, 0.1% sodium benzoate, 0.1% saccharin, and food colorings.

### Intervention

All eligible patients diagnosed with gastrointestinal cancer who reported fatigue were referred to the researcher by an oncologist and were randomly assigned to two parallel groups. One group received 10 mL of honey syrup three times daily as the intervention, while the control group received 10 mL of placebo three times daily. The intervention lasted for four weeks. At baseline, participants completed an informed consent form, personal information sheets, and questionnaires including the Visual Analogue Fatigue Scale (VAFS)—a validated questionnaire employing a numerical scale ranging from zero to ten, where zero indicates the absence of fatigue and ten represents the most severe, intolerable level of fatigue; the Fatigue Severity Scale (FSS)—a questionnaire consisting of nine questions, five of which assess the severity of fatigue. Each question is scored on a scale from 1 to 7, where a score of 1 indicates complete disagreement and a score of 7 indicates complete agreement. A total score of 36 or higher suggests that the individual requires medical evaluation and treatment for fatigue; and the Cancer-Related Fatigue Scale (CFS)—which measures the patient's assessment of fatigue severity, including three subgroups: physical, emotional, and cognitive. It consists of 15 questions, each scored on a scale from 1 to 5. The total score ranges from 15 to 75. Scores of 15-35 indicate mild fatigue, 36-55 indicate moderate fatigue, and 56-75 indicate severe fatigue. They were instructed to report any new symptoms or abnormalities that arose during the study and were advised to continue their routine medications. Participants were informed that they could withdraw from the study at any time. At the end of four weeks, the questionnaires were re-administered, and the collected data were analyzed statistically by a statistician. To identify potential side effects, all patients were monitored biweekly by a physician. Patients were also asked to report any adverse events, particularly gastrointestinal issues and allergic reactions.

### Randomization and Blinding

In this study, the restricted randomization method of block randomization was used. Blocking helps balance the number of participants assigned to each study group. The randomization was performed using balanced block randomization into four blocks via computer. Each drug was labeled with a number from 1 to 120. The randomization process, including the use of computer-generated sequences and the implementation of allocation concealment through coded bottles prepared by a pharmacist, was designed to prevent predictability and ensure unbiased allocation.

Patients were divided into two groups for the trial: the intervention group (60 people) and the control group (60 people). Both groups were matched in terms of characteristics and baseline conditions. The control group was assigned the label "A", and the intervention group "B". These groups were then divided into six blocks of four, with arrangements including: (1) AABB, (2) BBAA, (3) ABAB, (4) BABA, (5) ABBA, and (6) BAAB. These blocks were randomly combined by a computer to form a chain of groups. Participants were placed into these groups in the order of enrollment.

For randomization, we used the Random Allocation Software, which can generate sequences through simple randomization or block randomization. To ensure allocation concealment, the process was blinded so that group assignments were unknown prior to participant enrollment.

Ma-ol-asal or placebo bottles were produced with identical shape and color. After coding with a random sequence, each sequence was recorded on a label that was affixed to the respective bottle. Participants received these labeled bottles. Both Ma-ol-asal and placebo were prepared by the pharmacist in the laboratory, stored in identical bottles, and only the pharmacist knew the codes. The bottles were then provided to the research team. This process ensured that both the researchers and participants remained blinded to group assignments throughout the study.

### Endpoints and Assessment

The primary endpoint of the study was to assess the effect of Ma-ol-asal on alleviating fatigue in patients with gastrointestinal cancer. The secondary endpoints included evaluating patient satisfaction and monitoring any adverse effects associated with Ma-ol-asal consumption in this patient group. Questionnaires assessing fatigue were administered at baseline (week 0) and post-intervention (week 4), and all these instruments have previously undergone validity testing. These included: VAFS; a numeric scale from 0 to 10, where 0 indicates no fatigue and 10 represents the most severe, intolerable fatigue. Scores of 4 or higher suggest the need for medical treatment for fatigue [16][17]. FSS; comprising 9 questions, with 5 questions primarily assessing fatigue severity. Each question is scored from 1 (strongly disagree) to 7 (strongly agree), and the total score is obtained by summing all responses. Higher scores indicate greater fatigue severity [18]. CFS; this scale evaluates patients’ perceived fatigue across physical, emotional, and cognitive domains. It consists of 15 questions, each scored from 1 (none) to 5 (very severe). The total score ranges from 15 to 75, with scores of 15-35 indicating mild fatigue, 36-55 moderate fatigue, and 56-75 severe fatigue [19]. Participants were asked to rate their satisfaction with the treatment using a numerical scale ranging from 0%, indicating the lowest satisfaction, to 100%, indicating the highest satisfaction. Any side effects reported by the participants were systematically documented as secondary outcomes on standardized blank sheets, which were provided to patients during the recruitment phase to ensure comprehensive recording.

### Statistical Analysis

All statistical analyzes were performed by SPSS software (IBM Corp. IBM SPSS Statistics for Windows, Version 25.0. Armonk, NY). The presentation of data was based on the mean ± standard deviation. The comparison of the mean before and after each group was done with paired t-test and the comparison of the mean of two groups together was done by t-test. Statistically, P<0.05 was considered significant. Given the impact of baseline score differences on the examined parameters, covariance analysis (ANCOVA) was used to control for potential confounding effects.

## Results

**Figure-1:**
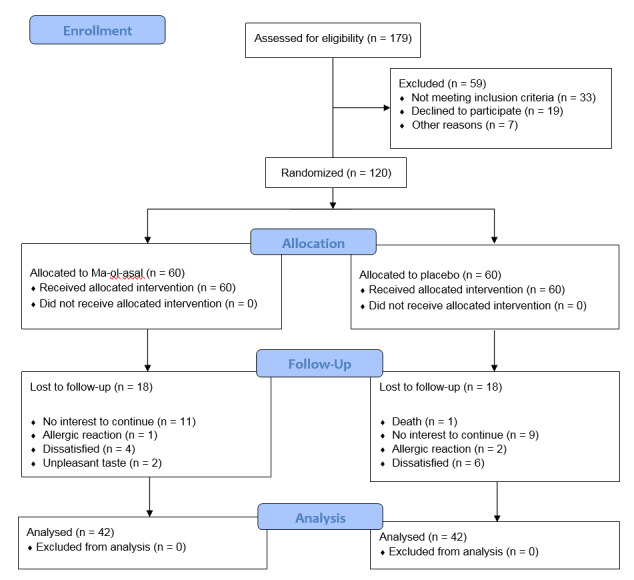
Figure 1. CONSORT flowchart

**Table:** Table1. Demographic Data of Participants

	**Intervention group ** **(Mean ± SD) **	**Placebo group ** **(Mean ± SD) **
**Age (year)**	59.43 ± 7.81	56.17 ± 11.26
**Height (cm)**	166 ± 13	168 ± 8
**Weight (Kg)**	67.7 ± 12.8	63.9 ± 13.4
**BMI**	25.16 ± 9.65	22.62 ± 3.82
**Hemoglobin level **	12.02 ± 1.37	12.43 ± 1.48
**Platelet count **	252898 ± 108704	269543 ± 126004
**WBC count**	6273 ± 3089	7140 ± 3479
	**n (%) **	**n (%) **
**Gender** Male Female	26 (61.9%) 16 (38.1%)	26 (61.9%) 16 (38.1%)
**Education** Illiterate Diploma or less More than diploma	15 (35.7%) 23 (54.8%) 4 (9.5%)	14 (33.3%) 22 (52.4%) 6 (14.3%)
**Type of cancer ** Esophagus Gastric Colorectal Liver Pancreas	2 (4.8%) 23 (54.8%) 16 (38.1%) 1 (2.4%) 0 (0.0%)	6 (14.3%) 10 (23.8%) 19 (45.2%) 6 (14.3%) 1 (2.4%)
**Blood pressure ** NO YES	33 (78.6%) 9 (21.4%)	35 (83.3%) 7 (16.7%)
**Underlying disease ** NO YES	40 (95.2%) 2 (4.8%)	37 (88.1%) 5 (11.9%)
**Smoking** NO YES	40 (95.2%) 2 (4.8%)	36 (85.7%) 6 (14.3%)

**SD:** Standard deviation; **cm:** centimeter; **Kg:** kilogram Statistical analysis is based on t-test / Chi-square test.

**Table:** Table2. Comparison of Fatigue Scale Scores in Cancer Patients Before and After Ma-ol-asal Syrup Intervention

	**Intervention group ** **(Mean ± SD) **	**Placebo group ** **(Mean ± SD) **	**P-value**	**Cohen d **
VAFS before after	5.07 ± 2.29 3.62 ± 2.24	5.19 ± 2.26 3.54 ± 2.11	0.811 0.857	**0.000**
P. value	**< 0.001**	**< 0.001**		
FSS before after	29.05 ± 12.24 25.07 ± 11.82	25.52 ± 9.72 24.02 ± 11.24	0.148 0.405	**0.091**
P. value	0.231	**0.009**		
CFS total before after	41.21 ± 6.37 39.60 ± 6.24	38.98 ± 7.10 37.55 ± 5.51	0.132 0.276	**0.35**
P. value	0.144	0.211		
CFS physical before after	19.86 ± 6.38 19.19 ± 5.86	18.36 ± 5.57 17.79 ± 5.06	0.254 0.531	**0.257**
P. value	0.447	0.469		
CFS affective before after	12.88 ± 2.95 12.4 5± 3.70	13.31 ± 2.88 12.19 ± 3.91	0.502 0.578	**0.069**
P. value	0.508	0.069		
CFS cognitive before after	8.55 ± 3.10 7.88 ± 2.67	7.31 ± 3.32 7.60 ± 2.50	0.081 0.880	**0.11**
P. value	0.276	0.597		
Diff VAFS	1.4524 ± 2.08599	1.5714 ± 1.92725	0.903	
Diff FSS	3.9762 ± 9.41584	1.5000 ± 8.00381	0.335	
Diff CFS	1.6190 ± 7.04325	1.4286 ± 7.29230	0.737	
Diff CFS physical	0.6667 ± 5.62515	0.5714 ± 5.06611	0.886	
Diff CFS affective	0.4286 ± 4.15635	1.1190 ± 3.87740	0.421	
Diff CFS cognitive	0.6667 ± 3.91163	-0.2857 ± 3.47314	0.206	

**SD:** Standard deviation; **VAFS:** Visual Analogue Fatigue Scale; **FSS:** Fatigue Severity Scale; **CFS:** Cancer-Related Fatigue Scale. Statistical analysis is based on t-test or paired t-test.

Before the completion of the trial, 35 patients withdrew from the study, and one patient died prior to the end of the study. Thus, the study was carried out on 42 patients in each group. No statistically significant differences were reported between the groups regarding demographic, type of cancer, and laboratory tests (P>0.05). Figure-1 illustrates the flow diagram of recruitment, group allocation, intervention, follow-up, and data analysis. Analysis of data from participants who completed the study showed that 32 (38.1%) were female and 52 (61.9%) were male. The mean age was 59.43 ± 7.81 years in the intervention group and 56.17 ± 11.26 years in the control group. All patients had gastrointestinal cancers: 35 (41.7%) with colorectal, 33 (39.3%) with gastric, 8 (9.5%) with esophageal, 7 (8.3%) with liver, and 1 (1.2%) with pancreatic cancer. The demographic characteristics are detailed in Table-1. Except for the variable of tumor type (P=0.018), no significant differences were observed between groups regarding baseline demographic variables such as age, hemoglobin level, body mass index, education level, or other factors.

In the intervention arm, the mean baseline VAFS score was 5.07 ± 2.29 out of 10, and in the placebo arm, it was 5.19 ± 2.26. At the end of the study, the mean VAFS score was very close in both groups (Table-2). The results showed that the baseline VAFS score significantly influenced within-group differences in both groups (P<0.001). However, post-intervention and follow-up assessments revealed no significant difference in average VAFS scores between groups (P=0.86).

For the FSS scale, the mean baseline score was 29.05 ± 12.24 out of 45 in the intervention group and 25.52 ± 9.72 in the placebo group. At the end of the study, mean scores were 25.07 ± 11.82 in the intervention group and 24.02 ± 11.24 in the placebo group. Covariance analysis indicated that no significant between-group differences was observed after intervention and follow-up in baseline or final FSS scores.

In the intervention group, the mean baseline CFS score was 41.21 ± 6.37 out of 75, while in the placebo group, it was 38.98 ± 7.10. At the end of the study, the mean CFS score was 39.60 ± 6.24 in the intervention group and 37.55 ± 5.51 in the placebo group. Covariance analysis indicated that no significant difference was observed between groups post-intervention.

Regarding the physical domain, baseline scores showed no significant effect (P=0.25). After adjusting for baseline scores, no significant difference was found between groups post-intervention. In the Affective domain, no significant difference was observed in the baseline and final scores. For the Cognitive domain, although baseline scores did not significantly confound the results, no significant difference between groups was found post-intervention. Overall, as summarized in Table-2, no significant differences in the mean scores of the assessed variables were observed after intervention.

Adverse events were reported by 47.6% (20 patients) in the intervention group and 47.6% (20 patients) in the placebo group. The most common adverse effects were abdominal pain, nausea, and vomiting, each reported by 13 patients. The most frequent adverse effect in the intervention group was nausea and vomiting (6 patients), while in the placebo group, it was abdominal pain (9 patients). Among the intervention group, 21% reported the treatment as not beneficial, whereas 28% of the placebo group shared this opinion. Additionally, in the intervention group, 35% rated the usefulness of the medication as moderate, 40% as good, and 2% as excellent; in the placebo group, these figures were 11%, 57%, and 2%, respectively.

## Discussion

The findings revealed that Ma-ol-asal syrup led to notable clinical improvements in patients' fatigue scores; however, no statistically significant difference was found between the two groups. Several possible explanations may account for the observed significant changes in both groups. These results could be due to the placebo effect associated with receiving an intervention or other aspects of participating in a clinical trial. Alternatively, they might reflect the natural course of the symptoms over time or stem from selecting a specific subgroup of patients with higher baseline fatigue levels (selection bias towards those experiencing above-average fatigue during screening). Nonetheless, it appears unlikely that these latter explanations played a substantial role in the observed improvement in fatigue [20].

Several confounding factors potentially contributing to fatigue—such as depression or other mood disorders, physical disability, tumor stage, and chemotherapy—were considered [21]. To minimize the effects of confounders and bias, in addition to randomization and predefined inclusion and exclusion criteria, only patients who developed fatigue after initiating chemotherapy and had no prior history of fatigue were included. However, complete randomization concerning all confounding variables may not have been achieved. To address this, the analysis focused on confirming baseline equivalence between groups through statistical tests. To address this, the analysis focused on confirming baseline equivalence between groups through statistical tests. Statistically, no significant differences were observed between the groups. Patients with advanced cancer generally have a poor prognosis, and the likelihood of fatigue worsening over time is higher than its improvement [20]. This was mitigated through the use of different fatigue scales for screening and the trial.

According to Table-2, based on the VAFS and CFS criteria, Ma-ol-asal was not more effective than placebo in improving fatigue related to cancer. Nevertheless, fatigue significantly improved in the intervention group. Interestingly, fatigue also improved in the placebo group. The finding that such similar results can be observed in a double-blind trial between placebo and active treatment is remarkable. Recent systematic reviews and meta-analyses suggest that placebo responses in clinical trials investigating anti-fatigue therapies related to cancer are significant and should be taken into account [22].

Using the FSS criteria (Table-2), Ma-ol-asal demonstrated a greater effect on fatigue than placebo, with a mean reduction of 5.90 points versus 2.40 points—a difference of 3.5 points. Although this difference was not statistically significant, it could be clinically meaningful. Thus, the substantial improvement observed in both arms of this trial is likely attributable to the placebo effect, especially given the highly subjective nature of fatigue, which is highly susceptible to placebo influences [20].

The placebo effect is a complex phenomenon with unclear mechanisms, possibly influenced by patient expectations, beliefs, the patient-provider relationship, natural disease variability, and regression to the mean [23]. This means that extreme measurements often move closer to the average over time [23][24]. Individual responses may vary based on factors like prior activity levels, comorbid symptoms, or age [25]. Research indicates there are associations between fatigue and responses to treatments, and future studies should focus on identifying which subgroups respond best to interventions. Additionally, biases such as the Hawthorne effect, where the attention from researchers improves symptoms, can also be associated with the placebo effect [23]. Generally, the placebo effect in clinical trials is reported to be between 30% and 40%, with some evidence suggesting this is increasing [26]. In the study, 59% of the placebo group reported treatment satisfaction, highlighting how a strong placebo effect can complicate the interpretation of therapeutic efficacy.

In 2019, Aguiar Junior and colleagues conducted a meta-analysis examining the efficacy of placebo in treating Cancer-Related Fatigue (CRF). This meta-analysis included 29 studies with 3,758 participants and found that 29% of patients receiving placebo experienced significant improvements in CRF. They concluded that placebo treatments have a meaningful and notable effect on CRF, which could inform future study designs for CRF management [27]. Our findings are consistent with those of Aguiar Junior and colleagues.

As previously mentioned, the placebo effect involves a fundamental role of the doctor-patient relationship. In this context, physicians are the same researchers who proposed the clinical trial. Patients’ beliefs and expectations about their physicians may influence their behavior, potentially reinforcing those beliefs and expectations. A very close relationship can develop between investigators and the study participants, leading to a strong commitment from some patients toward the anticipated outcomes [28], known as the Pygmalion effect.

An important finding in this study is the dropout rate in both groups, which appears to be primarily due to disease progression and side effects. The overall incidence and severity of adverse events during the trial were similar between the two groups, indicating that the dropout rate might have occurred randomly. The most frequent adverse event in the Ma-ol-asal group was nausea and vomiting, reported by six participants, while in the placebo group, it was abdominal pain, reported by nine participants. Although some side effects were expected due to the underlying malignant disease and tumor location, it is recommended that future studies include patients with other types of cancers outside the gastrointestinal tract.

Previous research has shown that attendance in a clinical trial and telephone follow-up of patients can be beneficial in managing fatigue and related symptoms in cancer patients. Expectation of improvement in fatigue as part of participating in a clinical trial may be due to the placebo/nocebo effect. Further investigation is necessary to clarify the role of expectancy in symptom reduction during clinical trial participation [29]. To our knowledge, this is the first clinical trial to evaluate the efficacy of Ma-ol-asal syrup in managing fatigue in patients with gastrointestinal cancer undergoing chemotherapy. Nevertheless, clinical trials have demonstrated the safety and efficacy of Ma-ol-asal in treating cystic fibrosis, asthma, chronic obstructive pulmonary disease, and polycystic ovary syndrome. No serious adverse effects have been reported in these studies [30][31][32][11].

Previously, multiple trials have been conducted to assess the effects of Iranian traditional medicine formulations on fatigue. For example, Heydarirad et al. investigated the effect of Nokhodāb—a traditional Iranian food—on fatigue in breast cancer patients receiving chemotherapy [33]. Although their study, like ours, evaluated the efficacy of a traditional remedy on CRF and used similar fatigue scales and follow-up methods, it differed in the intervention substance, sample size, and cancer types. Their results indicated that Nokhodāb could significantly reduce VAFS and FSS scores, unlike our findings. However, our study’s advantages include a larger sample size and soliciting participant feedback on the medication’s usefulness.

Another study by Mofid and colleagues examined the effects of royal jelly and processed honey on CRF among patients undergoing hormonal therapy, chemotherapy, and radiotherapy. Their results suggested that royal jelly and processed honey, compared to placebo, improved CRF [34]. In contrast, our study observed significant improvements in both arms, which diverges from Mofid et al.'s findings. Additionally, the baseline fatigue levels in our study were higher, as we used a cutoff score of 4 on the VAFS scale for screening. Another distinction—and a strength—of our study is the use of the CFS, a specialized questionnaire for assessing CRF, whereas Mofid et al. only employed the more general VAFS and FSS scales. Other advantages of our study include the larger sample size, the homogeneity of participants regarding disease type across groups, and the lower cost of Ma-ol-asal syrup and placebo compared to royal jelly and processed honey, reducing the economic burden on patients.

In another study, Heydarirad et al. examined the effect of Jollab—a saffron-based beverage—on CRF in patients with breast cancer undergoing chemotherapy [35]. The intervention material, cancer type, and sample size differed from our study. Their results indicated that Jollab significantly improved cognitive and physical fatigue in cancer patients, a finding that does not align with ours. The advantages of our study include a larger sample size and the assessment of participant satisfaction with the intervention. Future randomized controlled trials are recommended to compare the effects of Ma-ol-asal syrup and Jollab on fatigue in patients with gastrointestinal cancers.

Considering that the patients in our study were generally unwell, further research might focus on fatigue in early-stage cancer patients or those experiencing treatment-related fatigue, who may respond differently. Recently, a Phase 2 trial of methylphenidate was conducted for fatigue in women with early-stage breast cancer, supporting this notion [36]. In our study, nearly one-third of participants in the placebo group withdrew, which may indicate dissatisfaction. One limitation of this study is that fatigue measurements were only assessed at weeks 0 and 4, without continuous monitoring during the trial.

The primary strengths of this study include the relatively large sample size with adequate statistical power, a double-blind, randomized, placebo-controlled design, patient-reported outcomes, and a relatively homogeneous study population, which helps reduce variability among participants. As this is the first clinical trial investigating the efficacy of Ma-ol-asal syrup in managing fatigue among patients with gastrointestinal cancer, direct comparison with similar studies is limited. Additional studies with larger samples and refined methodologies are necessary to address this gap. Another limitation is the high attrition rate over the 28-day period. Given that significant clinical improvements in fatigue were observed in both arms without statistically significant differences, it is unlikely that the dropout rate significantly influenced the overall results. Furthermore, the use of subjective outcome measures and the short duration of the study are additional limitations.

## Conclusion

Our study provides important negative evidence, clearly demonstrating that Ma-ol-asal syrup does not outperform placebo in alleviating CRF. The absence of significant differences between the Ma-ol-asal group and the placebo group suggests that any improvements in fatigue scores across the VAFS, FSS, and CFS scales may not be attributable to the treatment itself.

Additionally, this finding aligns with previous reports on similar compounds, reinforcing the notion that certain treatments may not provide the anticipated benefits over placebo. The results call for a critical re-evaluation of treatment approaches for CRF, emphasizing the need for rigorous research to ascertain the efficacy of various interventions. Future studies should prioritize exploring alternative therapies that demonstrate clear advantages over placebo in managing symptoms effectively.

## Conflict of Interest

None.
